# A Multi-Fork Z-Axis Quartz Micromachined Gyroscope

**DOI:** 10.3390/s130912482

**Published:** 2013-09-17

**Authors:** Lihui Feng, Ke Zhao, Yunan Sun, Jianmin Cui, Fang Cui, Aiying Yang

**Affiliations:** School of Optoelectronics, Beijing Institute of Technology, Beijing 100081, China; E-Mails: kirt@bit.edu.cn (K.Z.); syn@bit.edu.cn (Y.S.); cueijianmin@bit.edu.cn (J.C.); cf@bit.edu.cn (F.C.); yangaiying@bit.edu.cn (A.Y.)

**Keywords:** quartz micromachined gyroscope, multi-fork, coriolis force, anisotropic etching

## Abstract

A novel multi-fork z-axis gyroscope is presented in this paper. Different from traditional quartz gyroscopes, the lateral electrodes of the sense beam can be arranged in simple patterns; as a result, the fabrication is simplified. High sensitivity is achieved by the multi-fork design. The working principles are introduced, while the finite element method (FEM) is used to simulate the modal and sensitivity. A quartz fork is fabricated, and a prototype is assembled. Impedance testing shows that the drive frequency and sense frequency are similar to the simulations, and the quality factor is approximately 10,000 in air. The scale factor is measured to be 18.134 mV/(°/s) and the nonlinearity is 0.40% in a full-scale input range of ±250 °/s.

## 1. Introduction

**Figure 1.**
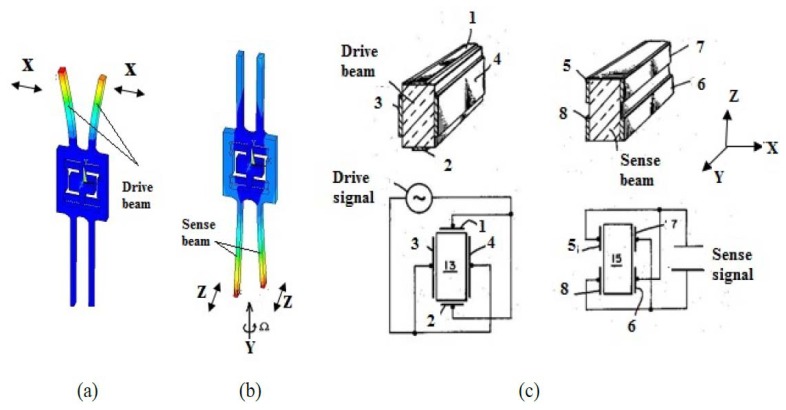
Vibration modes and electrode distribution of a traditional gyroscope. (**a**) Drive mode (**b**) Sense mode and (**c**) Electrode distribution.

The quartz gyroscope is an important sensor used in various civil and military applications [1–6]. For most quartz gyroscopes, the drive mode is the fork or beam's vibration in the x-axis orientation and the sense mode is the vibration in the z-axis orientation, while the input angular rate is in the y-axis orientation. The drive mode, sense mode and electrode distribution of a typical quartz gyroscope are shown in Figure 1a–c.Two parallel independent sense electrodes on each sidewall of the sense beam (in Figure 1c, parts 5 and 8 as well as 6 and 7 are separated) are required to gather the charges induced by the sense mode. However, the whole thickness of the quartz tuning fork is about several hundred micrometers, so performance of the sensors is affected by fabrication errors and it is difficult to deposit complicated electrode patterns [3,4]. Therefore, many researchers are working to explore a comprehensive approach to reduce the process difficulties and to improve the performance of the sensors [7–9]. In this paper, a type of z-axis gyroscope is introduced. It is fabricated using the quartz anisotropic wet etching process. Details about the structure design, fabrication process, readout circuit, and characterization of this kind of gyroscope are presented.

The organization of the paper is as follows: first a brief description of the principle and structure is provided in the Introduction section. Device modeling is discussed in Section 2. Theoretical analysis and simulation are introduced in Section 3. Fabrication and signal detection methods are described in Section 4, while experimental test results are presented in Section 5, and the summary and conclusions are given in the last section.

## 2. Design Concept

### 2.1. Structure of the Z-Axis Multi-Fork Gyroscope and Working Principle

**Figure 2.**
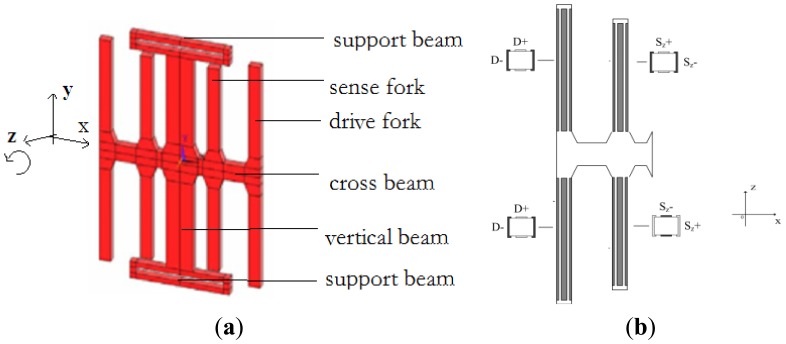
Structure and electrode distribution. (**a**) The structure of the gyroscope and (**b**) The electrode distribution of the left part.

The working principle of a quartz gyroscope is based on the Coriolis effect. In this paper, we present a z-axis gyroscope. The structure of the drive fork and sense fork are shown in Figure 2a. Four drive forks and four sense forks are arranged symmetrically. The electrode distribution of the left part of the gyroscope is shown in Figure 2b, while the right part is symmetrical to the left part.

**Figure 3.**
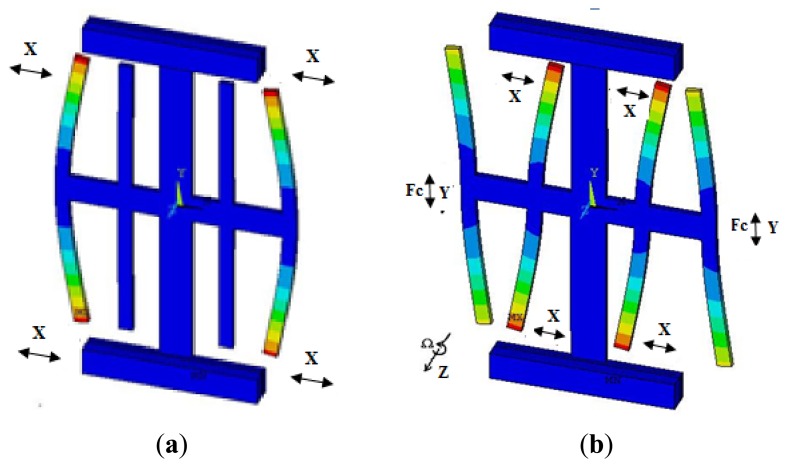
Vibration modes of the gyroscope. (**a**) Drive mode and (**b**) Sense mode.

When the drive forks are driven, a vibration is induced along the x-axis based on the inverse piezoelectric effect, while an opposite vibration occurs on the four drive forks in x-axis direction. The drive mode is shown in Figure 3a. When the angular rate is applied on the z-axis, the Coriolis force can make the cross beam move up or down leading to vibration in the sense mode as shown in Figure 3b. The bending of the sense forks in the x-axis can generate a charge because of the piezoelectric effect, which is proportional to the Coriolis force and detected by the sense electrode. The angular rate can be obtained by demodulating this charge signal.

Therefore, the following benefits can be determined when comparing the gyroscope presented in this paper with a traditional y-axis gyroscope:

1. The fabrication of the electrode is easy: it can sense the angular rate without complex electrodes.
2. The thickness of the wafer has little effect on the mode frequency; thus, the wafer can be made thinner, but the shock resistance should be considered.
3. The symmetry of this structure can suppress the effects of the linear acceleration and cross-coupling, *etc.*

In addition, when compared to other z-axis gyroscopes [7–9], the sensitivity will be greater due to the increase of the vibration amplitude of the sense forks.

### 2.2. Design Rules

**Figure 4.**
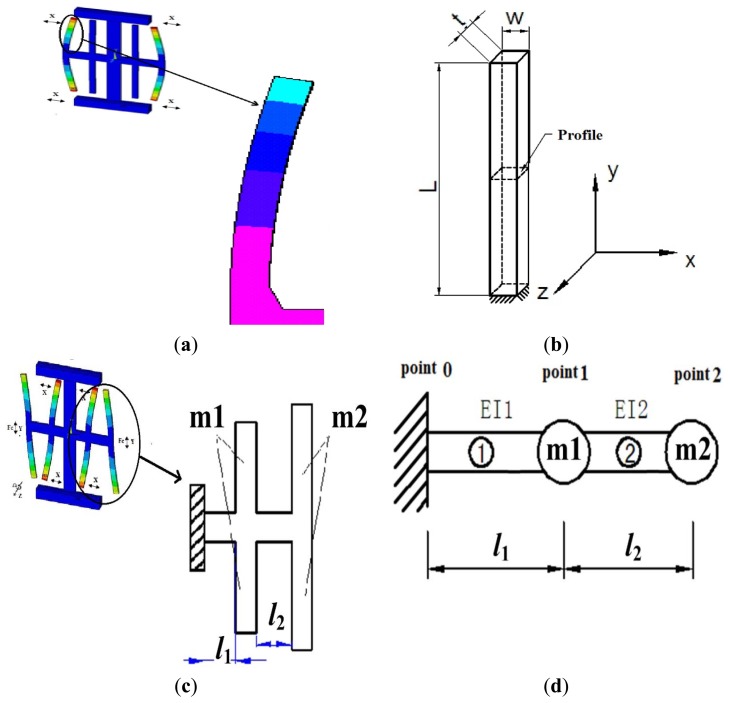
(**a**) One beam of the drive mode; (**b**) Simplified model of the drive fork; (**c**) Right side of the sense mode; (**d**) Simplified model of the right side in the sense mode.

The most basic design goal is to obtain the appropriate resonant frequency; One beam of the drive mode is shown in Figure 4a and a simplified driving fork is shown in Figure 4b. The modal coordinate Equation is expressed as [10]:

(1) $$\xi_{n}=\cos\text{h}(\mu_{n}y)-\cos(\mu_{n}y)-\alpha_{n}[\sin\text{h}(\mu_{n}y)-\sin(\mu_{n}y)]$$

where, *μ_n_* and *α_n_* can be obtained by:

(2) $$\cos\text{h}(\mu_{n}l)\cos(\mu_{n}l)\pm 1=0$$

(3) $$\alpha_{n}=[\cos\text{h}(\mu_{n}l)\pm \cos(\mu_{n}l)]/[\sin\text{h}(\mu_{n}l)\pm \sin(\mu_{n}l)]$$

From this equation, it is possible to express the resonant equation, as below:

(4) $$\omega_{i}=\sqrt{\frac{EI}{\rho Al^{4}}}{\left(\mu_{i}l\right)}^{2}$$

where E and ρ represent the Young's modulus and the density of the quartz, respectively. The sense mode includes the vibration of the cross beam, drive fork, and sense fork, it shown in Figure 4c. To simplify this model, it is considered that the sense fork and drive fork have the masses m1 and m2 (where m = density × volume), based on which the model can be expressed as in Figure 4d, where *l*_1_ and *l*_2_ represent the length of the left and right parts of the cross beam.

The Transfer Matrix Method [11,12] can be used solve the modal coordinate equations of this structure that have been separated into several limited units from a complex system. The dimension changes of the drive fork and sense fork can change the m1 and m2 values. The changes in m1, m2, *l*_1_ and *l*_2_ can cause a resonant frequency change in the sense mode. The different beam has four elements *x* = [*ω*, *θ*, *M*, *Q*]*^T^*, which are lateral displacement, cross section angle of declination, bending moment, and shear force respectively. The stiffness of the bending moment is *EI*.

Figure 4d shows that the sense beam can be considered as two point masses (m1, m2) and two beams ((1), (2)). We designate the quantities of the left and right of the mass by superscripts L and R, respectively.

First, considering the beam section from point 0 to point 1, the following equations can be derived [12]:

(5-a) $${\left\{\begin{array}{c} \omega \\ \theta \\ M\\ Q \end{array}\right\}}_{1}^{L}=\left[\begin{array}{cccc} 1 & l_{1} & \frac{l_{1}^{2}}{2EI} & \frac{l_{1}^{3}}{6EI}\\ 0 & 1 & \frac{l_{1}}{EI} & \frac{l_{1}^{2}}{2EI}\\ 0 & 0 & 1 & l_{1}\\ 0 & 0 & 0 & 1 \end{array}\right]*{\left\{\begin{array}{c} \omega \\ \theta \\ M\\ Q \end{array}\right\}}_{0}^{R}$$

Next, considering the point mass from the left of point 1 to the right of point 1, the following equations can be obtained:

(5-b) $${\left\{\begin{array}{c} \omega \\ \theta \\ M\\ Q \end{array}\right\}}_{1}^{R}=\left[\begin{array}{cccc} 1 & 0 & 0 & 0\\ 0 & 1 & 0 & 0\\ 0 & 0 & 1 & 0\\ m_{1}\omega^{2} & 0 & 0 & 1 \end{array}\right]*{\left\{\begin{array}{c} \omega \\ \theta \\ M\\ Q \end{array}\right\}}_{1}^{L}$$

From Equations (5-a) and (5-b), we can get the Equation (5-c):

(5-c) $${\left\{\begin{array}{c} \omega \\ \theta \\ M\\ Q \end{array}\right\}}_{1}^{R}=\left[\begin{array}{cccc} 1 & l_{1} & \frac{l_{1}^{2}}{2El_{1}} & \frac{l_{1}^{3}}{6E_{1}}\\ 0 & 1 & \frac{l_{1}}{El_{1}} & \frac{l_{1}^{2}}{2El_{1}}\\ 0 & 0 & 1 & l_{1}\\ {\text{m}}_{1}\omega^{2} & l_{1}{\text{m}}_{1}\omega^{2} & m_{1}\omega^{2}\frac{l_{1}^{2}}{2El_{11}} & m_{1}\omega^{2}\frac{l_{1}^{2}}{6El_{11}}+1 \end{array}\right]*{\left\{\begin{array}{c} \omega \\ \theta \\ M\\ Q \end{array}\right\}}_{0}^{R}$$

*x* = [*ω θ M Q*]*^T^*, Equation (5-c) can be simplified as:

$$x_{1}^{R}=G_{1}x_{0}^{R},$$

where:

(5-d) $$G_{1}=\left[\begin{array}{cccc} 1 & l_{1} & \frac{l_{1}^{2}}{2El_{1}} & \frac{l_{1}^{3}}{6El_{1}}\\ 0 & 1 & \frac{l_{1}}{El_{1}} & \frac{l_{1}^{2}}{2El_{1}}\\ 0 & 0 & 1 & l_{1}\\ {\text{m}}_{1}\omega^{2} & l_{1}m_{1}\omega^{2} & m_{1}\omega^{2}\frac{l_{1}^{2}}{2El_{1}} & m_{1}\omega^{2}\frac{l_{1}^{2}}{6El_{1}}+1 \end{array}\right]$$

Using the same method, the relationship from point 1 to point 2 can be written as $x_{2}^{R}=G_{2}x_{1}^{R}$ , where:

(5-e) $$G_{2}=\left[\begin{array}{cccc} 1 & l_{2} & \frac{l_{2}^{2}}{2EI_{2}} & \frac{l_{2}^{3}}{6EI_{2}}\\ 0 & 1 & \frac{l_{2}}{EI_{2}} & \frac{l_{2}^{2}}{2EI_{2}}\\ 0 & 0 & 1 & l_{2}\\ m_{2}\omega^{2} & l_{2}m_{2}\omega^{2} & m_{2}\omega^{2}\frac{l_{2}^{2}}{2EI_{2}} & m_{2}\omega^{2}\frac{l_{2}^{2}}{6EI_{2}}+1 \end{array}\right]$$

Using the boundary conditions of the beam, which are expressed in Equation (6):

(6) $$\begin{array}{l} \omega_{0}=0,\theta_{0}=0\\ M_{2}=0,Q_{2}=0 \end{array}$$

The state vector of the left point on the beam can be expressed as in Equation (7):

(7) $$X_{0}^{R}={\left[\begin{array}{llll} \omega_{0} & \theta_{0} & M & Q \end{array}\right]}^{T}={\left[\begin{array}{llll} 0 & 0 & M & Q \end{array}\right]}^{T}$$

Thus, Equation (8) is obtained:

(8-a) $$x_{1}^{R}=G_{1}x_{0}^{R}=\left[\begin{array}{c} M\frac{l_{1}^{2}}{2EI_{1}}+Q\frac{l_{1}^{3}}{6EI_{1}}\\ M\frac{l_{1}}{EI_{1}}+Q\frac{l_{1}^{2}}{2EI_{1}}\\ M+l_{1}Q\\ Mm_{1}\omega^{2}\frac{l_{1}^{2}}{2EI_{1}}+Q(m_{1}\omega^{2}\frac{l_{1}^{2}}{6EI_{1}}+1) \end{array}\right]$$

(8-b) $$x_{2}^{R}=G_{2}x_{1}^{R}=\left[\begin{array}{c} M(\frac{l_{1}^{2}}{2EI_{1}}+\frac{l_{1}l_{2}}{EI_{1}}+\frac{l_{2}}{2EI_{1}}+\frac{l_{2}^{3}}{6EI_{1}}m_{1}\omega^{2}\frac{l_{1}^{2}}{2EI_{1}})+Q(\frac{l_{1}^{3}}{6EI_{1}}+\frac{l_{1}^{2}}{2EI_{1}}+\frac{l_{1}l_{2}}{2EI_{2}}+\frac{l_{2}^{3}}{6EI_{2}}m_{1}\frac{\omega^{2}l_{1}^{2}}{6EI_{2}})\\ M(\frac{l_{1}}{EI_{1}}+\frac{l_{2}}{EI_{2}}+\frac{l_{2}^{3}}{2EI_{2}}m_{1}\omega^{2}\frac{l_{1}^{2}}{2EI_{1}})+Q(\frac{l_{1}^{2}}{2EI_{1}}+\frac{l_{1}l_{2}}{EI_{2}}+m_{1}\omega^{2}\frac{l_{1}^{2}}{6EI}+1)\\ M(1+l_{2}+m_{1}\omega^{2}\frac{l_{1}^{2}}{2EI_{1}})+Q(l_{1}+l_{2}(m_{1}\omega^{2}\frac{l_{1}^{2}}{6I_{1}}+1))\\ M(m_{2}\omega^{2}\frac{l_{1}^{2}}{2EI_{1}}+l_{2}m_{2}\omega^{2}\frac{l_{1}}{EI_{1}}+m_{2}\omega^{2}\frac{l_{2}^{2}}{2EI_{2}}+m_{1}m_{2}\omega^{4}\frac{l_{2}^{3}l_{1}^{2}}{12E^{2}I_{1}I_{2}}+m_{1}\omega^{2}\frac{l_{1}^{2}}{2EI_{1}})+Q(m_{2}\omega^{2}\frac{l_{1}^{2}}{6EI_{1}}+l_{2}m_{2}\omega^{2}\frac{l_{1}^{2}}{2EI_{1}}+m_{2}\omega^{2}\frac{l_{2}^{2}l_{1}}{2EI_{2}}+m_{1}m_{2}\omega^{4}\frac{l_{2}^{3}l_{1}^{2}}{36E^{2}I_{1}I_{2}}+m_{1}\omega^{2}\frac{l_{1}^{2}}{6EI_{1}}+m_{2}\omega^{2}\frac{l_{2}^{3}}{6EI_{2}}+1) \end{array}\right]$$

A system of homogeneous Equations can be obtained from the above result that are expressed in Equation (9):

(9) $$\begin{array}{l} M(1+l_{2}+m_{1}\omega^{2}\frac{l_{1}^{2}}{2EI_{1}})+Q(l_{1}+l_{2}(m_{1}\omega^{2}\frac{l_{1}^{2}}{6I_{1}}+1))=0\\ M(m_{2}\omega^{2}\frac{l_{1}^{2}}{2EI_{1}}+l_{2}m_{2}\omega^{2}\frac{l_{1}}{EI_{1}}+m_{2}\omega^{2}\frac{l_{2}^{2}}{2EI_{2}}+m_{1}m_{2}\omega^{4}\frac{l_{2}^{3}l_{1}^{2}}{12E^{2}I_{1}I_{2}}+m_{1}\omega^{2}\frac{l_{1}^{2}}{2EI_{1}})+Q(m_{2}\omega^{2}\frac{l_{1}^{2}}{6EI_{1}}+l_{2}m_{2}\omega^{2}\frac{l_{1}^{2}}{2EI_{1}}+m_{2}\omega^{2}\frac{l_{2}^{2}l_{1}}{2EI_{2}}+m_{1}m_{2}\omega^{4}\frac{l_{2}^{3}l_{1}^{2}}{36E^{2}I_{1}I_{2}}+m_{1}\omega^{2}\frac{l_{1}^{2}}{6EI_{1}}+m_{2}\omega^{2}\frac{l_{2}^{3}}{6EI_{2}}+1)=0 \end{array}$$

The determinant is assumed to be zero, and *ω_i_* (i is the order of the modal) can be obtained.

**Figure 5.**
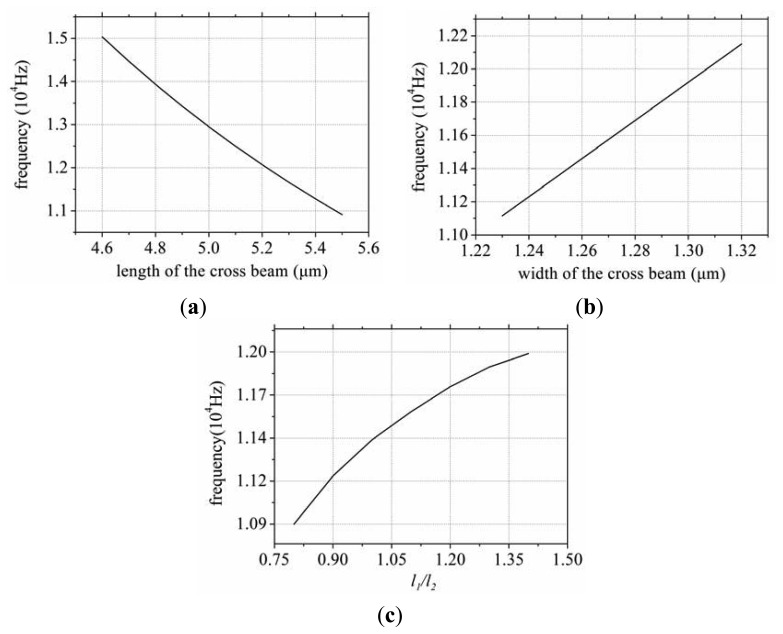
The relationship between frequency and various dimensions, (**a**) The length; (**b**) The width, and (**c**) The ratio of the *l_1_:l_2_*.

The results calculated in MATLAB are shown in Figure 5a–c, where *E* = 1/*S*_22_, *m* = *ρ* × *l* × *w* × *t*, $I=\frac{w\times t^{3}}{12}$.

From the above analysis, it is possible to draw a couple of conclusions.

1. As the length of the cross beam increases the sense mode frequency will decrease. As the width increases the sense mode frequency will increase. As the *l*_1_:*l*_2_ ratio increases the sense mode frequency will increase.
2. As the distance between the drive fork and the sense fork decreases, the sense mode frequency will become higher.

## 3. Simulation

### 3.1. Structure Simulation

**Figure 6.**
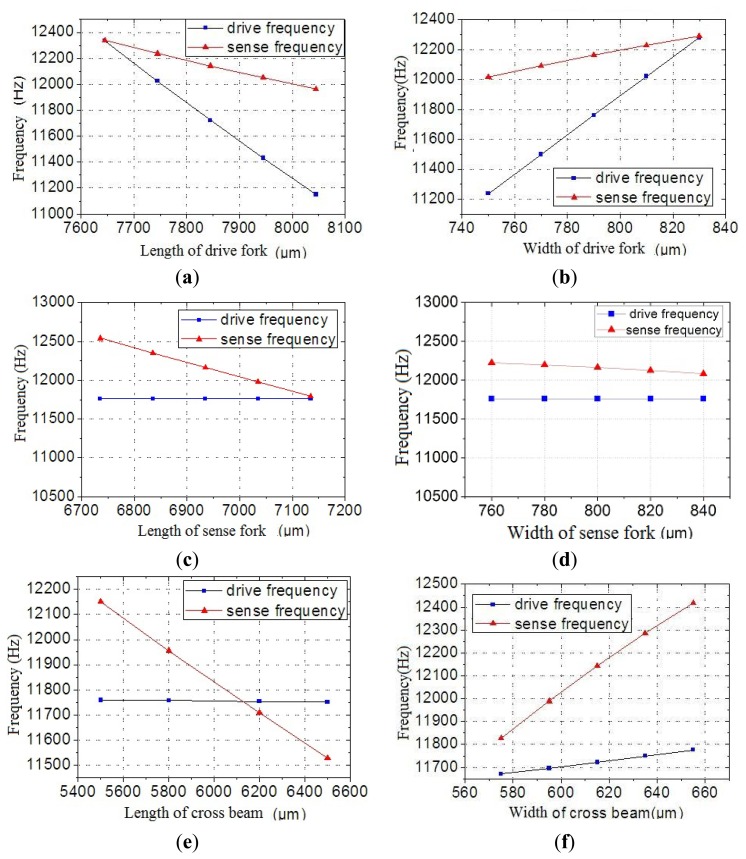
The effect of different fork dimension on the frequency. (**a**) Frequencies versus length of drive fork; (**b**) Frequencies versus width of drive fork; (**c**) Frequencies versus length of sense fork; (**d**) Frequencies versus width of sense fork; (**e**) Frequencies versus length of cross beam, and (**f**) Frequencies versus width of cross beam.

Finite element method (FEM) analysis is used to perform modelling and simulation. The frequency change with different dimension of the fork is simulated as shown in Figure 6a–f.

It can be seen from Figure 6a,b that the dimension change will affect the drive mode frequency and sense mode frequency. The drive mode is more sensitive to the dimension change than the sense mode. Figure 6c,d show that the dimension changes in the fork affects the frequencies. A change in the sense length has no influence on the drive frequency, but it changes the equivalent mass and sense frequency. In Figure 6e,f the results of the change in the cross beam dimensions on the frequency are shown. The dimension changes of the cross beam have a greater effect on the sense mode frequency than on the drive mode frequency.

**Figure 7.**
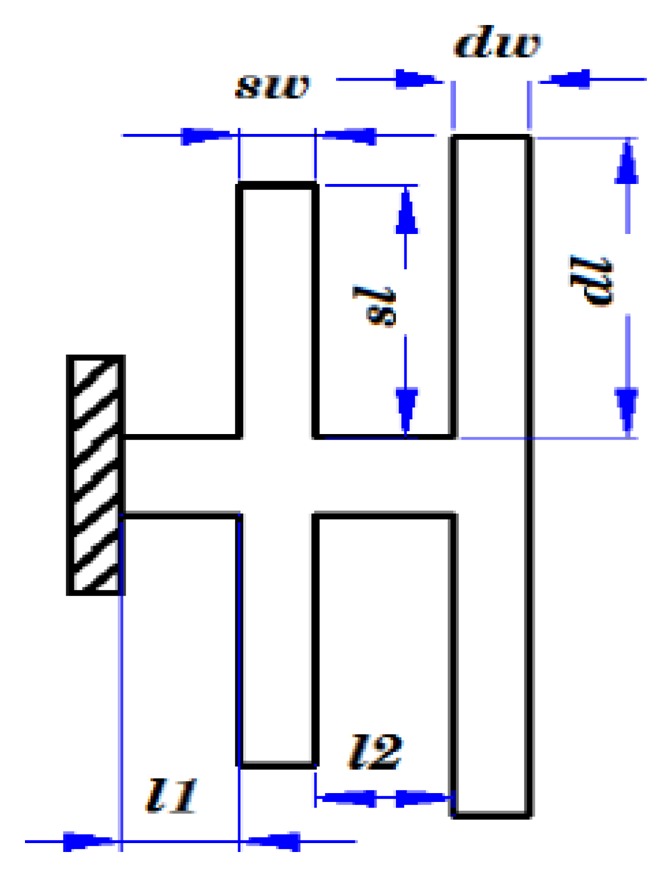
The dimensions of the chip.

These results can be used to design the dimensions of a structure. From the simulation, the dimensions of the chip are set as shown in Table 1 and Figure 7.

From the above analysis, the following conclusions can be drawn:

1. The drive mode frequency is determined by the drive fork dimensions.
2. Sense mode frequency is dominated by the width and length of the cross beam.
3. Changes in the drive fork and sense fork will change the equivalent mass, and affect the frequency of the sense mode.
4. The position of the sense fork will change the frequency of the sense mode.

### 3.2. Sensitivity of the Quartz Gyroscope

To obtain the highest sensitivity, the sensitivity can be expressed by Equations (10-a) and (10-b), in which *ω_r_*=*ω_d_* [13]:

(10-a) $$S=\frac{2\omega_{d}\xi_{x0}}{\omega_{s}^{2}\sqrt{{\left(1-\frac{\omega_{d}^{2}}{\omega_{s}^{2}}\right)}^{2}+{\left(\frac{\omega_{d}}{\omega_{s}Q_{d}}\right)}^{2}}}$$

(10-b) $$\phi_{s}=\arctan\left(\frac{\omega_{d}\omega_{s}}{Q_{s}(\omega_{s}^{2}-\omega_{d}^{2})}\right)$$

where, *S* is the sensitivity amplitude, *ϕ*_s_ is the sensitivity phase, *ω_r_* is the drive mode frequency and *ω_d_* is the drive voltage signal frequency, *ξ_x_*_0_ is the drive displacement, and *ω_s_* is the sense mode frequency.

**Figure 8.**
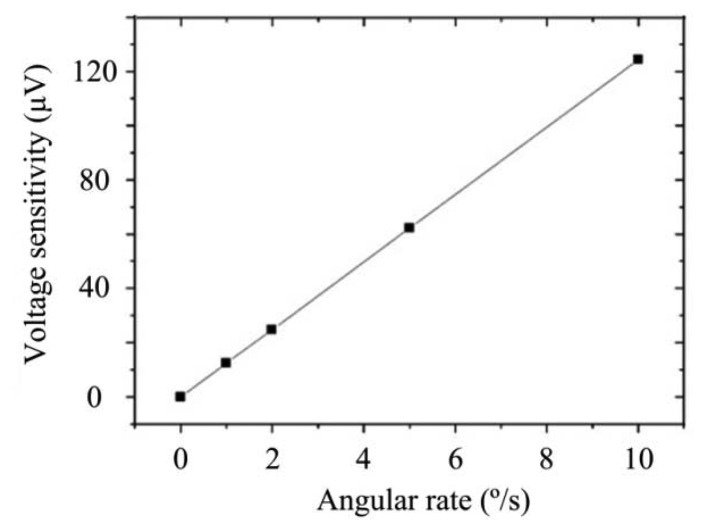
Simulation of voltage sensitivity.

The output of the gyroscope is the charge signal; so, the result can be simulated in ANSYS software. The voltage sensitivity result is shown in Table 2 and Figure 8, from which it is possible to determine the charge sensitivity, where the amplitude is 0.1022 fC/(°/s) and the phase is 179.795°.

## 4. Fabrication and Signal Detection

### 4.1. Fabrication Process

**Figure 9.**
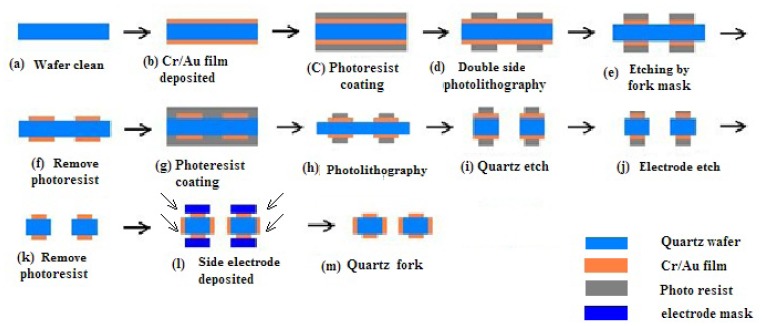
Fabrication process.

**Figure 10.**
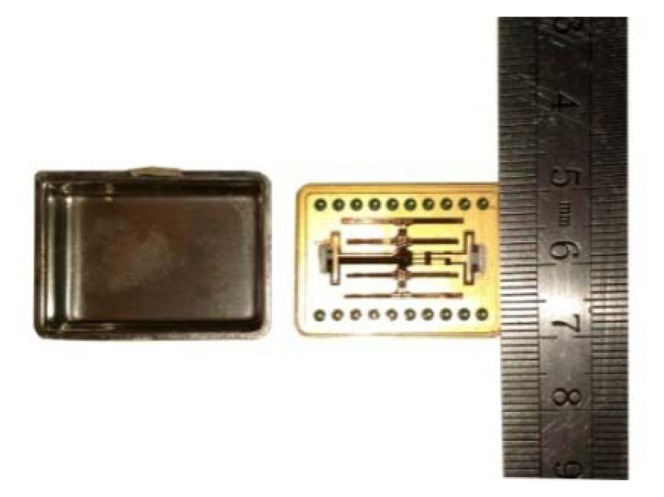
Packaged device.

The fabrication process is shown in Figure 9 [14]. The prototype gyroscope is fabricated using a surface polishing z-cut quartz wafer with a thickness of 500 μm. Two masks are prepared for the quartz fork figure and the electrode figure, called the quartz fork mask and the electrode mask respectively. The quartz gyroscope can be realized by the quartz anisotropic wet etching process. After the quartz wafer is cleaned (Figure 9a), Cr/Au films are deposited on the double surface, in which Cr film is used to add the adhesive force (Figure 9b). Double side photolithography and etching are used to make the Cr/Au mask, and the quartz fork mask is used during the photolithography (Figure 9c–f). Then double side photolithography method is used again to form the electrode pattern. The electrode mask will be used (Figure 9g,h) during the photolithography. A mixture of HF (40 wt % aqueous solution) and NH_4_F (40 wt % aqueous solution) (concentration ratio of the HF and NH_4_F is 1:2) is used to etch the quartz at 50 °C for 48 h. Long etching time can give rise to good etch result, while it is limited by the Cr/Au films adhesive force on the quartz wafer (Figure 9i). After the Cr/Au films are etched to form the electrode pattern, photoresist is removed (Figure 9j,k). Then side electrodes are deposited by vacuum sputtering coating with an angle (Figure 9l,m). Finally, the structure is mounted on a pedestal with epoxy and electrically connected to the surrounding electronics with Au wire bonding. The whole fabricated device is shown in Figure 10.

### 4.2. Impedance Testing

**Figure 11.**
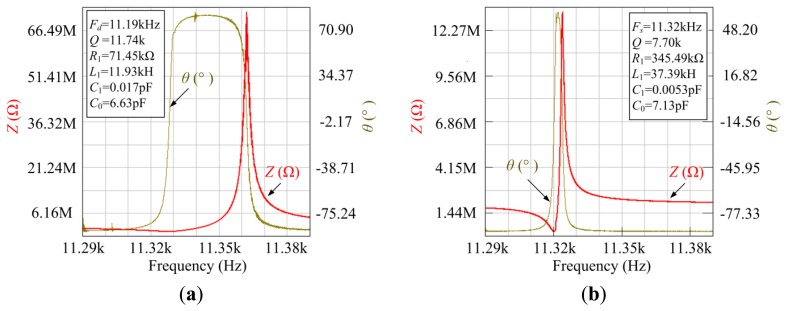
Impendence testing. (**a**) Impedance characteristic of drive mode and (**b**) Impedance characteristic of sense mode.

From Figure 11, it can be seen that the drive mode frequency *F_d_* and the sense mode frequency *F_s_* are measured to be 11.185 kHz and 11.320 kHz, which are similar to the theoretical analysis and simulation results. The Q factor of the drive mode and the sense mode is calculated to be 11,735 and 7,697 without vacuum packaging. The values of the drive mode and sense mode are shown in Table 3.

### 4.3. Signal Detection

**Figure 12.**
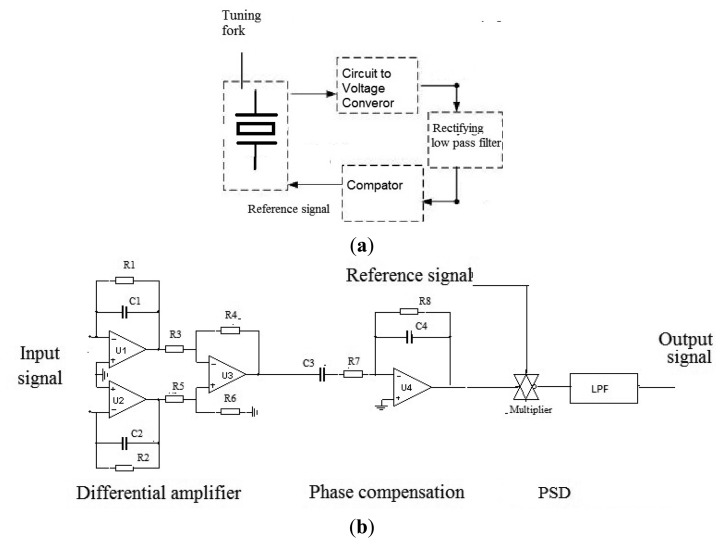
Signal detection process; (**a**) Drive part and (**b**) Detect part.

The signal detection method is shown in Figure 12 [15]. The differential amplifier is used to eliminate common mode noise. The optimal phase relationship between reference and input signals can be obtained in the phase compensation; while, the angular rate signal is demodulated using phase sensitive demodulators (PSD).

### 4.4. Performance Testing

**Figure 13.**
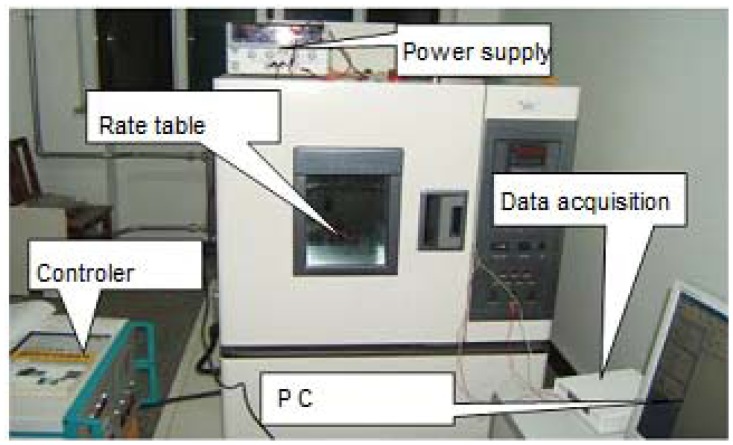
Testing system.

**Figure 14.**
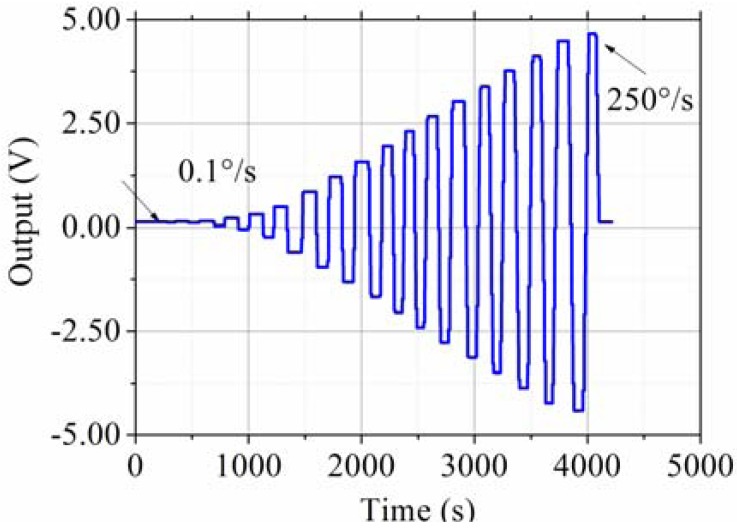
Output with different input angular rate with ±0.1 °/s, ±0.5 °/s, ±1 °/s, ±5 °/s, ±10 °/s, ±20 °/s, ±40 °/s, ±60 °/s, ±80 °/s, ±100 °/s, ±120 °/s, ±140 °/s, ±160 °/s, ±180 °/s, ±200 °/s, ±220 °/s, ±240 °/s, ±250 °/s.

**Figure 15.**
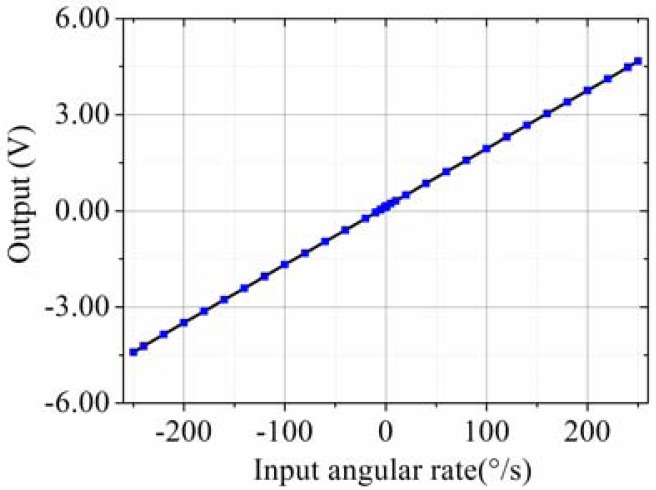
Statistical output with different input angular rate.

**Figure 16.**
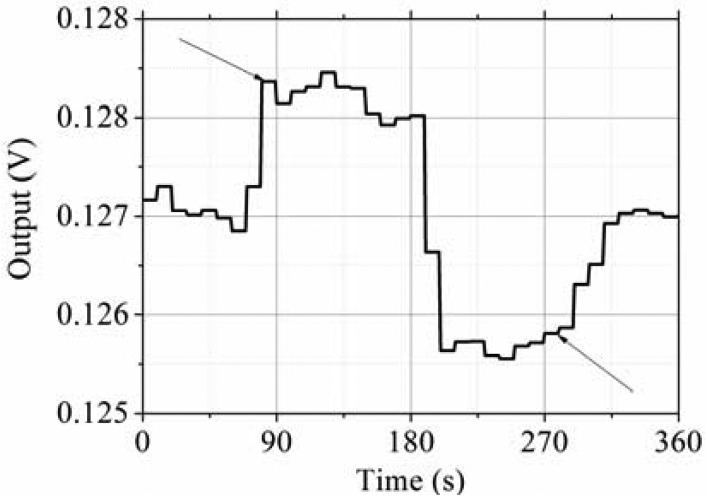
Sensitivity test.

The testing system is shown in Figure 13. The prototype is put on the rate table, the data is obtained by the data acquisition device at a sampling rate of 10 times the bandwidth, and the data is averaged to one value per second. The different input rates were from ±0.1 °/s to ±250 °/s, and the output voltages are recorded. The measured scale factor was 18.137 mV/(°/s) and the nonlinearity was 0.40% under a full-scale input range of ±250 °/s. The test results are shown in Figure 14 and statistical results are shown in Figure 15. The output with ±0.05 °/s angular rate input in shown in Figure 16, showing that the sensitivity is higher than 0.05 °/s.

## 5. Conclusions

In this paper, the modeling, simulation, fabrication, and performance characterization of a kind of multi fork z-axis quartz gyroscope are presented. With the proposed scheme, the fabrication process on the lateral electrode can be simplified compared to traditional quartz gyroscopes. Benefiting from the multi fork design, a higher sensitivity than possible with some other z-axis quartz gyroscopes is obtained. The structure is etched from a z-cut quartz wafer. The drive mode frequency is measured to be about 11 kHz and the quality factor is about 10,000 in air at atmospheric pressure, indicating the gyroscope can function in air without the need for a vacuum package. The experimentally obtained scale factor is 18.137 mV/(°/s) and the nonlinearity is 0.40% in the range of ±250 °/s. The sensitivity is higher than 0.05 °/s at the bandwidth of 50 Hz.

## Figures and Tables

**Table 1.** The dimensions of the chip (μm).

**Item**	**Value**	**Item**	**Value**
Length of drive fork	7,030	Width of drive fork	790
Length of sense fork	6,400	Width of sense fork	800
*l_1_*	1,230	*l_2_*	1,420
Thickness of fork	500		

**Table 2.** Simulation data with different angular rates.

**Angular rate (°/s)**	**Amplitude of Voltage Sensitivity (V)**	**Phase of Voltage Sensitivity (°)**
0	—	—
1	1.243 × 10^−5^	−0.207
2	2.485 × 10^−5^	−0.204
5	6.214 × 10^−5^	−0.203
10	1.243 × 10^−4^	−0.203

**Table 3.** Values of the drive mode and sense mode of the fork.

	**Frequency (kHz)**	***Q* Factor**	**Static Capacitance *C*_0_ (pF)**	**Dynamic Capacitance *C*_1_ (pF)**	**Dynamic Resistor *R*_1_ (kΩ)**	**Dynamic Inductance *L*_1_ (kH)**
Drive mode	11.185	11,735	6.631	0.017	71.450	11.931
Sense mode	11.324	7,697	7.131	0.005	345.485	37.387
